# The Impact of Sugar-Sweetened Beverage Taxes by Household Income: A Multi-City Comparison of Nielsen Purchasing Data

**DOI:** 10.3390/nu14050922

**Published:** 2022-02-22

**Authors:** Abigail R. Barker, Stephanie Mazzucca, Ruopeng An

**Affiliations:** Brown School, Washington University, St. Louis, MO 63130, USA; arbarker@wustl.edu (A.R.B.); smazzucca@wustl.edu (S.M.)

**Keywords:** SSB taxation, health policy, purchasing behavior, low income, equity

## Abstract

Due to the role that sugar-sweetened beverages (SSBs) play in the obesity epidemic, SSB taxes have been enacted in the United States in the California cities of Albany, Berkeley, Oakland, and San Francisco, as well as in Boulder, Philadelphia, and Seattle. We pooled five years of Nielsen Consumer Panel and Retail Scanner Data (2014–18) to examine purchasing behaviors in and around these cities that have instituted SSB taxes. We included households that were either subject to the tax during the study period or were in surrounding areas within the same state. The goal was to test for the differential impact of SSB taxes by income level and type of tax. Multivariate analyses of beverage purchases found that (1) there is a dose–response relationship with the size of the SSB tax; (2) the Philadelphia tax, which is the only one that includes low-calorie beverages, is associated with greater reductions in SSB purchases and an increase in bottled water purchase; and (3) approximately 72% of the tax is passed through to consumers, but this does not vary by income level of the household. Few income-related effects were detected. Overall, our findings suggest that the Philadelphia model may be the most effective at encouraging healthy habits in beverage choice.

## 1. Introduction

Rates of obesity and diabetes in the United States continue to climb, reaching all-time highs of 42.4% of adults (in 2017–18) and 10.2% (in 2013–16), respectively [1,2]. Explanations for these trends include many lifestyle changes, technological advances, and policy changes, many of which have made certain foods, e.g., energy-dense foods, significantly cheaper in real terms. Energy-dense foods, often laden with added sugars, contribute relatively more calories than essential nutrients such as fiber, vitamins, and minerals.

By identifying sugar-sweetened beverages (SSBs) as a primary source of added sugars in the Western diet, large-scale epidemiologic studies have substantiated the relationship between SSB consumption and childhood/adult obesity [3,4,5]. A meta-analysis of prospective cohort studies also suggests that there may be a dose–response relationship between SSB consumption and obesity, type 2 diabetes, hypertension, and all-cause mortality [6]. The World Health Organization (WHO) recommends that added sugar be less than 10% of daily total caloric intake, and additional health benefits may be attained if further reduced to 5% [7]. These daily recommendations can be easily exceeded by consuming a 500 mL single-serve bottle of SSB.

Thus, to address the obesity epidemic, some policy makers have turned to the concept of “junk food” taxes, particularly taxes on SSBs, to discourage purchase and thereby consumption. Fiscal policies to reduce the consumption of SSBs have been recommended by the WHO since 2015; such policies have already been adopted by more than 50 countries and territories around the world [8]. Studies have shown these initiatives to be effective in doing so [9,10,11,12], and much evidence links improvement in diet quality in general [13,14,15], through shifting away from energy-dense foods toward nutrient-dense foods to reduce obesity and diabetes prevalence. Some studies examine actual purchasing data at the aggregate or household level, usually for one taxed location at a time, while many others take a simulation approach. One study estimated that a penny-per-ounce SSB tax would reduce consumption by 15% and prevent 2.4 million diabetes person-years [6]. The first evidence of an association between SSB taxation and weight-related outcomes in adolescents has emerged from Mexico, where a tax has been in effect since 2014 [16].

Evidence from economics literature indicates that in general, sustained price changes (of the type a tax or subsidy can produce) are more likely to alter households’ consumption patterns significantly than would short-term price fluctuations due to seasonality and other market forces, as people are slow to seek out substitutes, form new habits, and develop new preferences [17,18]. In particular, low-income households face budget constraints that make them more sensitive to price increases [19].

Thus, even though emerging evidence suggests that SSB taxation may be an effective policy tool, it is crucial to understand the longer-term potential of taxation in terms of behavior change and the differential impact on low-income households [20]. Studies of purchasing in Oakland [21] and Seattle [22] found that the effect of the tax persisted two years afterward. As people switch away from SSBs subject to a tax, what will they purchase instead? How can the specific details of the tax (e.g., the size of the tax and the beverage subtypes targeted by the tax) influence consumer behavior? Some evidence suggests that consumers do indeed switch to non-taxed beverages [22,23] as well as engaging in cross-border shopping [24]. The latter, a particular problem for taxes at the city level, would tend to dampen the effect of the tax. A study of the Philadelphia tax found a greater reduction in SSB purchasing in low-income neighborhoods, although individual income data were not available [25]. A recent systematic review found that evidence regarding the distributional impact of SSB taxes is limited, with only 27% of included studies considering the issue, and mixed in the sense that 67% of those found a favorable impact on low-income groups [26].

To test for various changes in purchasing by household income, we will focus attention on the beverage purchases made by Nielsen households over time, with and without being subject to a tax, by income level. We hypothesize that low-income households may reduce purchasing by a greater amount in the presence of a tax, that a low-calorie beverage tax will increase the reduction in SSB purchases, and that a larger tax will have a greater impact. Significant reductions in SSB purchase may be accompanied by significant increases in the purchase of other beverages. Finally, while we expect the majority of the tax is likely to be passed through to consumers, as numerous other studies have found [21,27,28], we also hypothesize that this effect may be more pronounced for low-income consumers, who may have less flexibility on where to shop and fewer available substitutes that allow them to avoid the tax.

## 2. Materials and Methods

### 2.1. Study Population

This project pools five years of NielsenIQ panel and scanner data (2014–18) to examine purchasing behaviors in and around cities in the United States that have SSB taxes. During this time, SSB taxes were enacted in the California cities of Berkeley (March 2015), Albany (April 2017), Oakland (July 2017), and San Francisco (January 2018), as well as in Boulder, Colorado (July 2017), Philadelphia, Pennsylvania (January 2017), and Seattle, Washington (January 2018). The data are from a longitudinal consumer and market survey representing 54 major markets and updated annually [29]. Nielsen consumer panelists use in-home scanners to record all purchases from any outlet intended for personal, in-home use. We selected households either subject to the tax during the study period or living in surrounding areas within the same state; therefore, all Nielsen respondents living in Nielsen’s market regions within Northern California, Colorado, Pennsylvania, or Washington are included. We merged household data, which contains detailed demographic information including household size and indicator variables for the presence of children by age group, income category, and zip code, with purchasing behaviors using trip codes.

### 2.2. Taxation Measures

With reference to information from Healthy Food America [30], households were coded as belonging to a taxed zip code or not; based upon the trip purchase date, their purchases were further coded as subject to an SSB tax or not. In the case of the Philadelphia tax, low-calorie beverages were also taxed; this difference was also coded. Trip-level beverage purchasing data for one year prior and one year post-SSB tax implementation were then summarized by (1) calculating the number of ounces purchased in each of twelve beverage categories, using detailed product-level files, (2) aggregating to household totals in the untaxed and taxed periods, and (3) expressing these values as daily ounces purchased per household member. The twelve beverage categories were: carbonated soft drinks (CSDs), low-calorie carbonated soft drinks, bottled water, milk, fruit juices, fruit drinks, non-carbonated soft drinks, tea, coffee, beer, wine, and liquor. Because the taxing cities had different criteria for defining “sugar sweetened,” we focused on the first two categories for the purposes of detecting overall changes due to the tax. Most of the beverages categorized as “fruit drinks” and “non-carbonated soft drinks” were subject to taxation in most cities, but the Nielsen categorization of beverages did not allow us to definitively include these groups as being subject to the tax. Therefore, we analyze them separately. See Appendix A for additional details on the files used, the merging and coding process, and the subsampling criteria.

### 2.3. Income Measures

The key questions of interest were reducing SSB purchases, switching behavior, and whether these might differ according to income level. Because the SSB taxation in Northern California is concentrated in very high-income parts of the state, and in general that the cost of living in the different geographies included in this study is quite variable, we adjusted household income data for cost-of-living differences using a county-level index [31]. Cost-of-living-adjusted income was converted to a per person measure, i.e., income per household member, and a categorical variable for income being above/below the median in each state was created.

### 2.4. Statistical Analysis

We estimated a mixed regression model using SAS PROC HPMIXED (SAS Enterprise Guide 7.4; Cary, NC, USA) to explain the dependent variable, daily ounces of beverage purchases per household member. Independent variables were the SSB tax (measured in cents per ounce), Philadelphia’s low-calorie SSB tax indicator, and above/below median adjusted income per household member. Household ID was modeled as a random effect nested within a state; considerable state-level variation in SSB purchases motivates this choice [32]. Multivariate analyses were performed to obtain the main effects of the SSB tax and to test for changes in ounces of beverages purchased due to the inclusion of income variables.

A similar model was employed to estimate the change in prices of SSBs paid by consumers before and after taxes were imposed, also known as the “pass-through,” of SSB taxes of various sizes, using the average price paid per ounce of beverage as the dependent variable. While it is well established that the majority of the tax is typically passed through to consumers, the hypothesis here was that low-income households might be particularly vulnerable to the tax, i.e., the pass-through rates might differ for below- and above-median income households, perhaps due to differing access to grocery stores.

## 3. Results

This study included 529 households subject to an SSB tax in the seven cities listed above (Table 1), with two tiny samples (8 and 9 households each) in Boulder and Berkeley. A total of 669 households in taxed locations contributed data in the pre- or post-periods, while 8305 households in untaxed areas contributed data in the pre- or post-periods.

Table 2 shows that the basic model, Model A, does not detect a significant effect of SSB taxation on CSD purchase except in Philadelphia, where low-calorie CSDs were also taxed. Inclusion of income variables to adjust the intercept and slope in Model B found that higher-income households purchased fewer ounces of CSDs at baseline, but the tax effect on the two different income groups was not shown to be statistically different. Similarly, in Model C, accounting for other household demographics, we find that the presence of children in the household is strongly associated with lower CSD purchasing. Still, there is no statistically significant difference in their response to the tax. This correction in Model C improves the overall fit of the model such that we identify a significant relationship between the size of the tax in cents and CSD purchasing, with a reduction of 0.327 ounces per household per day per cent of tax. Thus, locations with 1.5, 1.75, or 2 cent taxes per ounce have larger overall associations between their taxes and CSD purchasing than locations with 1 cent per ounce tax.

Using similar regression methods and the specification of Model C, we next examined purchasing data for other beverages, including low-calorie CSDs, water, fruit drinks, fruit juices, tea, and milk. The results are reported in Table 3, where the main substitution effects created by the taxes are indicated in bold type. There is a statistically significant increase in bottled water purchases (0.767 ounces per household member per day per cent of tax, *p* < 0.001) in Philadelphia; since Philadelphia’s tax is 1.5 cents per ounce of SSBs, this translates to an increase of 1.15 ounces per household member per day. We did not detect a statistically significant reduction in low-calorie CSDs even in Philadelphia (−0.216 ounces per household member per day per cent of tax, *p* = 0.196), but we did detect reductions in the purchase of fruit drinks, which are generally also subject to tax in all locations. Note that the model found statistically significant shifts in the intercept in all models for the presence of children 0–12 in the household. Since purchases are normalized by the number of household members in the household, this adjustment may be viewed as a correction for the conflating of adults and children that occurs in that step.

In the models for CSD purchase (Table 2), higher-income households were associated with a reduced overall purchasing. This effect is not present and is sometimes reversed for the beverage purchases shown in Table 3. Higher-income households were statistically more likely to purchase low-calorie CSDs, bottled water, and fruit juice. Only in the purchase of bottled water did we find a significant effect of the tax that varied by income: households with income above the median had a decreased response to the tax in terms of bottled water purchase (−0.461 ounces per household member per day).

Finally, we examined the change in prices of CSDs paid by consumers before and after taxes were imposed. The hypothesis was that low-income households might be particularly vulnerable to the tax, i.e., the pass-through rates might differ for below- and above-median income households, perhaps due to differing access to grocery stores. Table 4 shows that this was not the case in this study: the income effect is insignificant. In untaxed areas, the average price per ounce was 4.7 cents, and, in general, approximately 72% of the tax was passed onto the consumer.

## 4. Discussion

This is one of the first studies to combine information about SSB and other beverage purchasing, with and without SSB taxation, across multiple cities using a single household-level data source. It is consistent with one other multi-city analysis, which used a different data source and analytic method, and also found that SSB purchasing reduction was concentrated in Philadelphia [33]. It is the first such study to investigate differential effects by household income and differences in switching behavior that might occur due to different taxation details (i.e., the size and applicability of the tax). The tax itself, measured in cents per ounce of beverage, was significantly associated with decreases in purchasing. While there were differences in initial purchasing patterns by income, we did not find a statistically different response to the tax in terms of SSB purchasing (either CSDs or fruit drinks) between lower- and higher-income households.

We found one difference in our analysis of bottled water purchasing, which suggested that lower-income households might be more likely to switch to bottled water than higher-income households in response to an SSB tax. This finding was similar to results based on a study of SSB and bottled water purchases in France [34] and in Tonga [35]. While it is possible that more households generally are purchasing bottled water over time, this result was specific to Philadelphia’s tax structure. There were no significant differences in purchasing SSBs (either CSDs or fruit drinks) or other beverages among households with children under 13. Even though there were no differences in how these SSB purchasing behaviors changed due to the tax, it is worth noting that because higher-income households consumed fewer ounces per day per household member to start, they do bear less of the burden of paying the tax.

A key takeaway of this research is that the Philadelphia tax, which encompasses low-calorie CSDs, had more sizeable and more significant effects on SSB purchasing than the other taxes on a per-cent-of-tax basis. All models of SSB purchase showed that while an SSB tax will discourage SSB purchasing, adding a low-calorie tax provides an additional disincentive by eliminating the option of switching to a low-calorie version of a beverage to avoid the tax. Furthermore, it seems that this tax design does promote the purchase of bottled water as a substitute, which is particularly encouraging for those who view the SSB tax as a tool that can promote healthy behavior. It may be even more impactful among lower-income households, which suggests that it targets those whose chronic disease burden is the most elevated due to dietary influence. It is also worth noting that the Philadelphia tax also achieves reductions in artificially sweetened beverages, which is important due to concerns raised that these “diet” beverages are actually associated with worse diet quality and may lead to weight gain and corresponding health risks [6,36].

These results, consistent with other work [37], aligns with the policy discussions regarding the best ways to use SSB revenues, as subsidies for healthy alternatives such as fruits and vegetables are frequently mentioned. One simulation study found that the tax burden of the poor would be mitigated by such an option, although it also noted that nutritional intake might not differ substantively [38]. Efforts to encourage substitution within the category of beverages may yield greater caloric changes—although incentivizing fruit and vegetable consumption remains a critical nutritional goal.

There are several limitations to this work. First, the observational study design using available Nielsen panel and scanner marketing data resulted in some households being observed only in the pre-tax period or only in the post-tax period, which further limits an already small sample size. Nielsen participants are not randomly selected, and the fact that they live in cities that chose to tax themselves on SSB purchases may create additional selection bias. Furthermore, only five cities in four states contributed data. These facts may limit generalizability and almost certainly result in fewer statistically significant results than we might have obtained with data collected explicitly for this purpose. Nielsen data do not contain detailed information about the exact numbers of adults and children in the household, making it difficult to interpret the findings in models that controlled for the presence of children in the household, since we could not establish per-adult or per-child data. Furthermore, the assignment of taxed status was made at the household level according to the zip code of residence. Still, it is quite possible that some households engaged in cross-border shopping and that this may have increased after the tax. If true, this would imply an understatement of the impact of the tax if it were to be implemented nationwide in the U.S. Finally, beverages in the Nielsen data were coded according to the categories mentioned above, but there are some individual products not considered CSDs that may be taxed depending on their added sugar content (e.g., coffees, teas); however, due to the volume of unique products, we did not assign each product a taxed/untaxed status.

## 5. Conclusions

Despite some limitations, this work provides evidence of the potential power of a broad-based tax on all sweetened drinks, regardless of the type of sweetener added. It also verifies that there is a significant dose–response relationship between the size of the tax and the amount of impact. A broad-based tax of 1 cent per ounce or more is associated with greater degrees of purchasing reduction in the beverage categories targeted by public health advocates, i.e., soft drinks and fruit drinks, while at the same time incentivizing the purchase of bottled water as a substitute. Policy makers taking this approach in the future may want to consider a tax of at least 1.5 cents/ounce on all sweetened beverages, and they may consider using some of the revenues generated to provide direct subsidies to the types of beverages being encouraged as substitutes, e.g., bottled water and unsweetened flavored waters and teas.

## Figures and Tables

**Table 1:** Household Counts in Study Sample.

Location	Size of Tax (cents/oz.)	Number of Households with Data Present in Pre-Tax Period	Number of Households with Data Present in Post-Tax Period
Berkeley	1	9	9
Albany, Oakland, San Francisco	1	215	175
Boulder	2	9	8
Philadelphia	1.5	236	220
Seattle	1.75	117	117
Total households in taxed locations		586	529
Northern California (excl. Berkeley)	−	940	923
Northern California (excl. Albany, Oakland, San Francisco)	−	927	784
Colorado (excl. Boulder)	−	1449	1426
Pennsylvania (excl. Philadelphia)	−	2877	2857
Washington (excl. Seattle)	−	1322	1262
Total households in untaxed locations		7515	7252

Includes 446 households in taxed locations with data in both pre- and post-periods and includes 6462 households in untaxed locations with data in both pre- and post-periods. Northern California households taxed in one city were removed as controls for other cities. Although reported separately in this table, they are combined into one state indicator in multivariate models. excl, excluding.

**Table 2:** Multivariate Models of Carbonated Soft Drink Purchasing by Study Households.

Variable	Model A	Model B	Model C
Intercept	**2.008** (1.916, 2.100)	**2.092** (1.977, 2.207)	**2.342** (2.215, 2.468)
SSB tax in cents	−0.104 (−0.327, 0.120)	−0.256 (−0.546, 0.035)	**−0.327** (−0.629, −0.025)
SSB tax in cents × Pennsylvania	**−0.403** (−0.647, −0.158)	**−0.523** (−0.808, −0.238)	**−0.597** (−0.898, −0.295)
Income above median		**−0.170** (−0.309, −0.030)	**−0.300** (−0.442, −0.158)
SSB tax in cents × income above median		0.272 (−0.061, 0.605)	0.323 (−0.018, 0.663)
Children 0–12 in household			**−0.953** (−1.155, −0.750)
SSB tax in cents × children 0–12 in household			0.296 (−0.285, 0.877)

Bold values are significant at the 0.05 level. SSB, sugar-sweetened beverages.

**Table 3:** Multivariate Models of Other Beverages Purchased by Study Households.

Variable	Low-cal CSDs	Bottled Water	Tea
Intercept	1.795 ***	1.494 ***	2.096 ***
**SSB tax in cents**	**−0.175**	**0.162**	**−0.393** **
**SSB tax in cents × Pennsylvania**	**−0.216**	**0.767** ***	**−0.273**
Income above median	0.474 ***	0.202 **	0.097
SSB tax in cents × income above median	0.238	−0.461 *	−0.017
Children 0–12 in household	−0.955 ***	−0.708 ***	−1.122 ***
SSB tax in cents × children 0–12 in household	0.098	−0.602	0.386
**Variable**	**Fruit Drinks**	**Fruit Juice**	**Milk**
Intercept	0.850 ***	0.816 ***	1.093 ***
**SSB tax in cents**	**−0.164** *	**−0.047**	**−0.135**
**SSB tax in cents × Pennsylvania**	**−0.232** **	**0.002**	**−0.120**
Income above median	0.044	0.138 ***	0.046
SSB tax in cents × income above median	0.141	−0.100	0.017
Children 0–12 in household	−0.120 *	−0.295 ***	−0.410 ***
SSB tax in cents × children 0–12 in household	−0.009	−0.052	−0.008

Bold and bold values indicate the main substitution effects due to the tax(es). Significance is indicated by *** (*p* < 0.001), ** (*p* < 0.01), or * (*p* < 0.05). SSB, sugar-sweetened beverages; CSDs, carbonated soft drinks.

**Table 4:** Average CSD Prices Paid by Study Households.

	Estimate	*p*-Value
Intercept	0.0471	<0.0001
SSB tax in cents	0.0072	<0.0001
Income above median	0.0006	0.4702

SSB, sugar-sweetened beverages.

## Data Availability

Nielsen data are proprietary and are only available to researchers through an application process. As no new data were created or analyzed in this study, data sharing is not applicable to this article. This work constitutes the researchers’ own analyses calculated (or derived) based in part on data from Nielsen Consumer LLC and marketing databases provided through the NielsenIQ Datasets at the Kilts Center for Marketing Data Center at The University of Chicago Booth School of Business. The conclusions drawn from the NielsenIQ data are those of the researchers and do not reflect the views of NielsenIQ. NielsenIQ is not responsible for, had no role in, and was not involved in analyzing and preparing the results reported herein.

## Appendix A

Detailed Information about the Construction of Study Sample 1. For years 2014–18, all scanner data related to beverage purchases was requested. Nielsen categories were combined into the following categories: CSDs, low-calorie CSDs, water, fruit drinks, fruit juices, non-carbonated soft drinks, milk, tea, coffee, beer, wine, and liquor. Files contained product information at the Universal Product Code (UPC) level and were used to categorize each UPC as one of the 12 beverage types. These are the “beverage files”. 2. Purchases files, UPC-level files indexed by trip code, contained information about the quantity purchased and price paid for each item. These files were merged by UPC with the beverage files to obtain “beverage purchase files”. 3. The most recent Products file, a UPC-level file that contains information about sizes and multi-packs, were used to convert each beverage purchase into a total number of ounces. (Teabags were assumed to generate an 8-ounce beverage each.) These files were merged with the beverage purchase files to create “beverage ounces purchased files”. 4. The UPC-level beverage ounces purchased files were summarized at the trip level to create “beverage ounces per trip files”. 5. Trips files provide trip-level data including the household code and the date of the purchase. These files were merged with the beverage ounces per trip files to create “beverage ounces per trip by household files”. 6. Panelists files contain demographic information about each household, including its zip code. Panelist data were merged by zip code with a manually created file containing SSB taxation information, including size of tax and effective date of tax, at the zip code level. This created a “panelist tax status file”, which was then reduced to contain observations in Northern California, Colorado, Pennsylvania, and Washington. 7. The combined 2014–18 panelist tax status file for the included states was merged with the combined 2014–18 beverage ounces per trip by household files, resulting in a trip-level summary file indexed by household that contained the date of purchase and the SSB tax status of the zip code of the household and the total ounces and average price paid for each of the 12 beverage types. 8. Trips were retained only if they fell within a defined pre-tax or post-tax period. Relative to the implementation date of each tax, a trip was retained if it occurred between 1 and 12 months before or 1 and 12 months after the implementation date. 9. California households that were taxed in one city were excluded from the comparison group for the other city. 10. The first and last dates of trips in the pre- and post-periods were calculated, resulting in a number of days the household was actively observed in the sample. Purchases were normalized to a daily equivalent, which corrected for the issue that some taxes’ pre- and post-periods spanned multiple survey years, resulting in some households that were only observed for partial years. Household size, a variable from the Panelists file, was also used to normalize the purchasing data. The final purchasing variables used were measured in ounces per household member per day. 11. The original income variable, also from the Panelists file, was a categorical variable. The midpoint of each income bracket was used as a continuous variable, divided by household size, and the variable was then adjusted using a county-level cost-of-living index. Zip-level data were assigned to the most likely county in cases were zip codes crossed county borders.
